# A new treefrog from Cordillera del Cóndor with comments on the biogeographic affinity between Cordillera del Cóndor and the Guianan Tepuis (Anura, Hylidae, *Hyloscirtus*)

**DOI:** 10.3897/zookeys.809.25207

**Published:** 2018-12-19

**Authors:** Santiago R. Ron, Marcel A. Caminer, Andrea Varela-Jaramillo, Diego Almeida-Reinoso

**Affiliations:** 1 Museo de Zoología, Escuela de Biología, Pontificia Universidad Católica del Ecuador, Av. 12 de Octubre y Roca, Aptdo. 17-01-2184, Quito, Ecuador Pontificia Universidad Católica del Ecuador Quito Ecuador

**Keywords:** Biodiversity, *
Colomascirtus
*, Ecuador, *H.larinopygion* group, Peru, prepollical spine, phylogeny

## Abstract

The *Hyloscirtuslarinopygion* group is a clade of 16 species of large hylids that inhabit cascading Andean streams. They have brown coloration that, in most species, contrasts with bright marks. Herein morphological and genetic evidence is used to describe a new species of the group from Cordillera del Cóndor, a sub-Andean mountain chain that has phytogeographic affinities with the Guianan Tepuis. The new species is characterized by dark-brown coloration with contrasting bright orange flecks and by the presence of an enlarged and curved prepollex protruding as a spine. The new species is closely related to *H.tapichalaca* and an undescribed species from the southern Andes of Ecuador. The genetic distance between *H.hillisi***sp. n.** and its closest relative, *H.tapichalaca*, is 2.9% (gene 16S mtDNA). Our phylogeny and a review of recently published phylogenies show that amphibians from Cordillera del Cóndor have close relationships with either Andean or Amazonian species. Amphibians do not show the Condor-Guianan Tepuis biogeographic link that has been documented in plants.

## Introduction

*Hyloscirtus*Peters 1882, is a genus of 37 species of treefrogs distributed from Costa Rica to the Andes of Bolivia, Colombia, Ecuador, Peru, and Venezuela (AmphibiaWeb 2018; Frost 2018). They reproduce along streams and share, as a synapomorphy, the presence of wide lateral fringes on fingers and toes (Faivovich et al. 2005 but see Coloma et al. 2012). A well-supported clade within *Hyloscirtus* is the *Hyloscirtuslarinopygion* species group (Almendáriz et al. 2014; Coloma et al. 2012; Duellman and Hillis 1990; Rivera-Correa et al. 2016). It is composed of 16 species characterized by large size (SVL < 60 mm) and gray or brown coloration that in many species contrast with bright marks. Species of this group were transferred to the genus *Colomascirtus* by Duellman et al. (2016). A recent phylogeny showed that the recognition of *Colomascirtus* rendered *Hyloscirtus* paraphyletic (Rojas-Runjaic et al. 2018). To maintain taxonomic stability, *Colomascirtus* was synonymized under *Hyloscirtus* by Rojas-Runjaic et al. (2018).

The *Hyloscirtuslarinopygion* group is composed of two well-supported clades that replace each other latitudinally with a small area of sympatry in central Ecuador (Almendáriz et al. 2014a). The northern clade is distributed in the Andes of central and northern Ecuador and southern Colombia; the southern clade is distributed in the eastern Andean slopes of central and southern Ecuador and northern Peru (Rivera-Correa et al. 2016). The southern clade is composed of three species: *H.condor* Almendáriz et al. 2014a, *H.tapichalaca* (Kizirian et al. 2003), and an undescribed species previously reported as *H.lindae* (Almendáriz et al. 2014a). *Hyloscirtusdiabolus*Rivera-Correa et al. 2016 is also a putative member of this clade (Rivera-Correa et al. 2016). The four species differ from species in the northern clade by having an enlarged prepollex with the shape of a spine that protrudes below the thumb (Almendáriz et al. 2014; Rivera-Correa et al. 2016). Recent fieldwork in Cordillera del Cóndor by a field team from the Museum of Zoology, Pontificia Universidad Católica del Ecuador, resulted in the discovery of an undescribed species of the southern clade which also shares a spine-shaped prepollex. Cordillera del Cóndor is a sub-Andean mountain chain with phytogeographic affinities to the Tepuis in the Guiana Region (e.g., Neill 2005). Herein we present morphological and genetic evidence to describe the new species and provide a new phylogeny for the genus *Hyloscirtus*. We also review recent amphibian phylogenies to explore the existence of biogeographic links between Cordillera del Cóndor and the Guianan Tepuis.

## Materials and methods

### DNA extraction, amplification, and sequencing

DNA was extracted from muscle or liver tissue preserved in 95% alcohol following standard phenol-chloroform extraction protocols (Sambrook et al. 1989). Standard polymerase chain reaction (PCR) was performed to amplify two mitochondrial genes (12S rRNA + tRNA^Val^ and 16S rRNA), using primers listed in Goebel et al. (1999), Heinicke et al. (2007), Hedges et al. (2008), and Heinicke et al. (2009) under standard protocols. PCR products were sequenced in both directions by Macrogen (Macrogen Inc., Seoul, Korea).

Sequences were edited and assembled with Geneious 10.2.3 software (Gene Matters Corp, Kearse et al. 2012). The obtained sequences were compared with those available in GenBank (http://www.ncbi.nlm.nih.gov/genbank/) for the *Hyloscirtuslarinopygion* and *bogotensis* groups (published by Almendáriz et al. 2014; Coloma et al. 2012; Darst and Cannatella 2004; Elmer and Cannatella 2008; Faivovich et al. 2004; Faivovich et al. 2005; Guayasamin et al. 2015; Rojas-Runjaic et al. 2018; Wiens et al. 2005; Wiens et al. 2006) (Table 1). For the outgroup we added sequences of *Aplastodiscusweygoldti*, *Bokermannohylacircumdata*, *Boanacrepitans*, *B.lundii*, *B.marianitae*, *B.riojana*, *Itapotihylalangsdorfii*, *Myersiohylakanaima*, and *Pseudacrisnigrita*.

**Table 1.** Genbank accession numbers for DNA sequences included in the phylogenetic analysis.

Species	Museum Number	GenBank Accession Number		Source
		12S	16S	
* Hyloscirtus alytolylax *	QCAZ 24376	JX155799	JX155826	Coloma et al. 2012
	QCAZ 24377	JX155798	JX155825	Coloma et al. 2012
* H. armatus *	KU 173222	AY819423	–	Wiens et al. 2005
	AMNH 165163	AY549321	AY549321	Faivovich et al. 2004
* H. callipeza *	UIS-A 5947	MG596780	MG596780	Rojas-Runjaic et al. 2018
* H. charazani *	AMNH 165132	AY843618	AY843618	Faivovich et al. 2005
* H. colymba *	SIU 6926	DQ380353	–	Wiens et al. 2006
	SIUC H-7079	AY843620	AY843620	Faivovich et al. 2005
* H. condor *	MEPN 14754	KF756939	KF756939	Almendáriz et al. 2014a
	MEPN 14758	KF756938	KF756938	Almendáriz et al. 2014a
* H. criptico *	QCAZ 43421	JX155812	JX155839	Coloma et al. 2012
	QCAZ 43422	JX155814	JX155841	Coloma et al. 2012
	QCAZ 45466	JX155813	JX155840	Coloma et al. 2012
*H.hillisi* sp. n.	QCAZ 68646	MH883792	MH883796	This study
	QCAZ 68647	–	MH883797	This study
	QCAZ 68648	MH883793	MH883798	This study
	QCAZ 68649	MH883794	MH883799	This study
	QCAZ 68651	MH883795	MH883800	This study
* H. jahni *	MHNLS 20318	MG596776	MG596776	Rojas-Runjaic et al. 2018
	MHNLS 20319	MG596777	MG596777	Rojas-Runjaic et al. 2018
	MHNLS 20324	MG596779	MG596779	Rojas-Runjaic et al. 2018
* H. japreria *	MHNLS 18888	MG596766	MG596766	Rojas-Runjaic et al. 2018
	MHNLS 19235	MG596769	MG596769	Rojas-Runjaic et al. 2018
	UIS-A 5496	MG596770	MG596770	Rojas-Runjaic et al. 2018
* H. larinopygion *	QCAZ 41826	JX155817	JX155844	Coloma et al. 2012
	QCAZ 45462	JX155818	JX155845	Coloma et al. 2012
* H. lascinius *	KU 181086	DQ380359	–	Wiens et al. 2006
	MHNLS 19163	MG596762	MG596762	Rojas-Runjaic et al. 2018
	MHNLS 19164	MG596763	MG596763	Rojas-Runjaic et al. 2018
* H. lindae *	QCAZ 41232	JX155821	JX155848	Coloma et al. 2012
	QCAZ 45342	JX155824	JX155851	Coloma et al. 2012
	QCAZ 45346	JX155822	JX155849	Coloma et al. 2012
	QCAZ 45463	JX155823	JX155850	Coloma et al. 2012
* H. mashpi *	MZUTI 614	KT279526	KT279511	Guayasamin et al. 2015
* H. pacha *	KU 202760	AY326057	AY326057	Darst and Cannatella 2004
	WED 53493	AY326057	AY326057	Darst and Cannatella 2004
* H. palmeri *	MZUTI 608	KT279549	KT279520	Guayasamin et al. 2015
	SIUC H-6924	AY843650	AY843650	Faivovich et al. 2005
* H. pantostictus *	QCAZ 45435	JX155820	JX155847	Coloma et al. 2012
	QCAZ 45438	JX155819	JX155846	Coloma et al. 2012
	KU 202732	AY326052	–	Darst and Cannatella 2004
* H. phyllognathus *	QCAZ 23938	JX155800	JX155827	Coloma et al. 2012
	QCAZ 32271	JX155802	JX155829	Coloma et al. 2012
	QCAZ 41032	JX155801	JX155828	Coloma et al. 2012
	KU 212119	DQ380369	–	Wiens et al. 2006
	MHNLS 20321	MG596772	MG596772	Rojas-Runjaic et al. 2018
	MHNLS 20325	MG596774	MG596774	Rojas-Runjaic et al. 2018
	QCAZ 42165	JX155806	JX155833	Coloma et al. 2012
	QCAZ 43654	JX155807	JX155834	Coloma et al. 2012
* H. psarolaimus *	QCAZ 27049	JX155808	JX155835	Coloma et al. 2012
	QCAZ 46095	JX155809	JX155836	Coloma et al. 2012
* H. ptychodactylus *	QCAZ 46030	JX155804	JX155831	Coloma et al. 2012
	QCAZ 46031	JX155805	JX155832	Coloma et al. 2012
* H. simmonsi *	KU 181167	DQ380376	–	Wiens et al. 2006
* H. staufferorum *	QCAZ 45962	JX155816	JX155843	Coloma et al. 2012
	QCAZ 45967	JX155815	JX155842	Coloma et al. 2012
* H. tapichalaca *	QCAZ 15083	JX155803	JX155830	Coloma et al. 2012
	QCAZ 16704	AY563625	AY563625	Faivovich et al. 2004
* H. tigrinus *	QCAZ 31550	JX155811	JX155838	Coloma et al. 2012
	QCAZ 41351	JX155810	JX155837	Coloma et al. 2012
*Hyloscirtus* sp.	MZUTI 3262	KT279503	KT279544	Guayasamin et al. 2015
	KU 202728	DQ380361	–	Wiens et al. 2006

Sequences were aligned using the Geneious extension MAFFT Multiple Alignment with the algorithm LINS-I (Katoh and Standley 2013). Alignments were imported into Mesquite (version 3.04; Maddison and Maddison 2018) for final visual adjustments. The final matrix included 2497 characters. The best partition strategy and best-fit model of nucleotide evolution for our data were obtained in PartitionFinder v.2.1.1 (Lanfear et al. 2012) under the corrected Akaike Information Criterion (AICc).

### Phylogeny

Phylogenetic relationships were inferred using maximum-likelihood and Bayesian inference. Maximum likelihood analysis were conducted with GARLI 2.0 (Zwickl 2006) using default values, except for the number of generations without topology improvement required for termination (genthreshfortopoterm = 30000) and the maximum number of generations to run and maximum search time (stopgen and stoptime = 5000000). A total of 40 independent searches were run, 20 started from random trees (streefname = random) and 20 from stepwise addition trees (streefname = stepwise). Likelihood values of the 40 searches were within 0.1 likelihood units of each other indicating that all searches converged on similar optimal trees. Support was assessed using 200 bootstrap pseudoreplicates. Bayesian phylogenetic analyses were carried out in MrBayes 3.2.6 (Ronquist et al. 2012). We made four parallel runs of the Metropolis-coupled Monte Carlo Markov for 20 million generations. Each run had five chains, sampled every 1000 generations and with a temperature of 0.1. Convergence into a stationary distribution was measured with software Tracer version 1.4 (Rambaut and Drummond 2007). The search was finished when the average standard deviations of split frequencies was < 0.05 between runs and ESS values were > 200 for all parameters. The consensus tree was generated after discarding 10% of the initial generations as burn-in. Bayesian analyses were carried out at Cipres Science Gateway (available at https//www.phylo.org; Miller et al. 2010).

Pairwise genetic distances between-species (uncorrected-*p*) were calculated with MEGA 5 (Tamura et al. 2011) for genes 16S (886 bp) and 12S (773 bp). Genetic distances for gene 16S are the most widely used standard to identify candidate species (e.g., Coloma et al. 2012; Fouquet et al. 2007; Janzen and Hallwachs 2011; Vieites et al. 2009).

### Morphology

Specimens of the new species were compared to published descriptions and alcohol-preserved specimens of the *Hyloscirtuslarinopygion* group from Museo de Zoología at Pontificia Universidad Católica del Ecuador, Quito (QCAZ). Examined specimens are listed as Appendix 1. Webbing formulae of hand and foot follow Savage and Heyer (1967) as modified by Myers and Duellman (1982). Morphological measurements were taken with digital calipers (± 0.01 mm) from specimens fixed in 10% formalin and preserved in 70% ethanol according to the methodology described in Duellman (1970). Measurements are: SVL (snout-vent length); HL (head length); HW (head width); ED (eye diameter); TD (tympanum diameter); TL (tibia length); FEL (femur length); and FL (foot length). Sex was determined by direct examination of gonads.

We also compared qualitative morphological characters between the new species and its closest relatives. Six characters were evaluated: (1) dorsal coloration; (2) ventral coloration; (3) marks on flanks and hidden surfaces of thighs; (4) iris coloration; (5) prepollex condition; and (6) in life, webbing coloration. Life coloration was obtained from color photographs.

## Results

### Phylogeny and genetic distances

**Figure 1.**
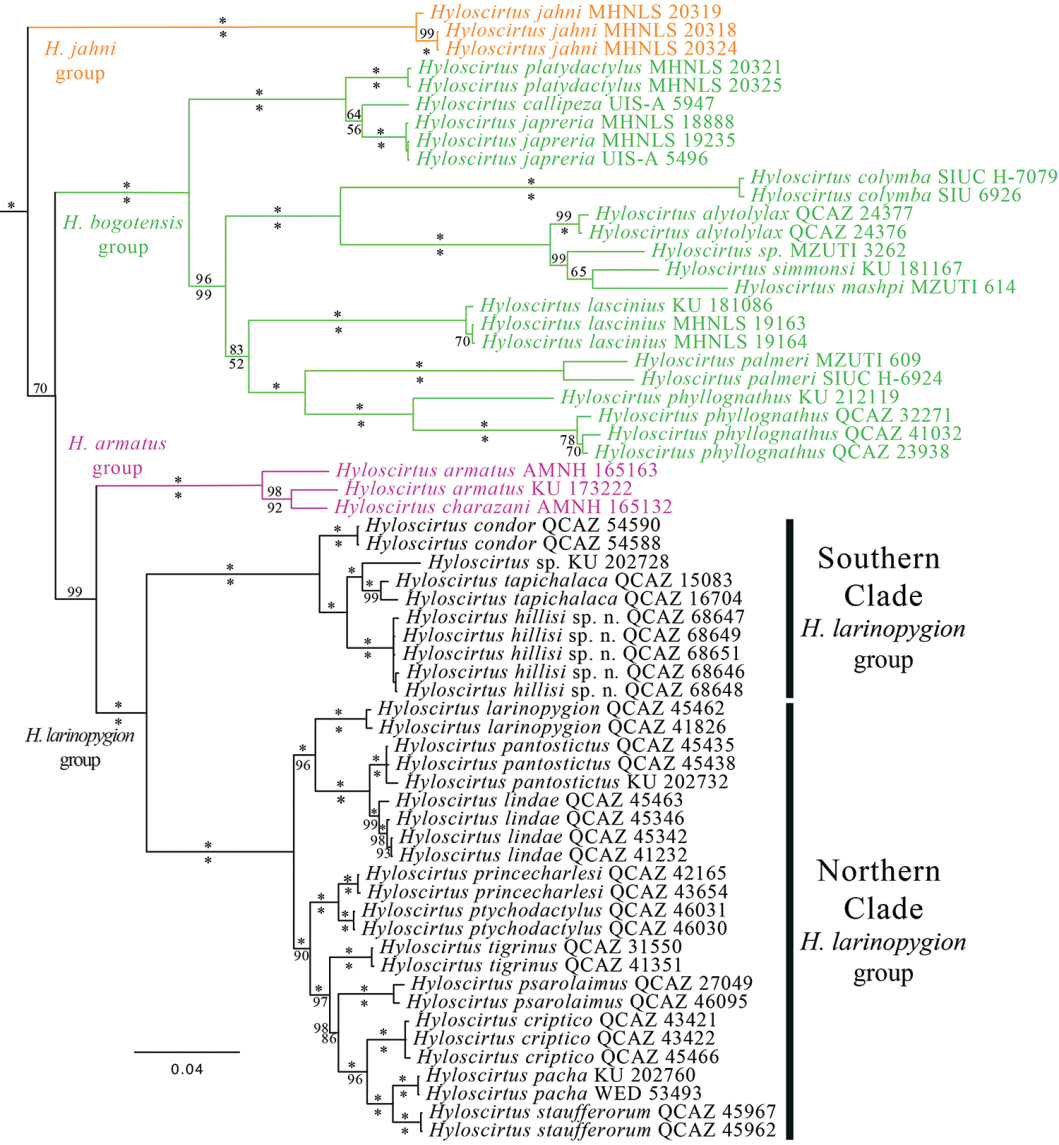
Strict consensus tree of *Hyloscirtus* species inferred with Bayesian inference. Museum numbers are shown for each sample. Bayesian posterior probabilities (pp × 100) are shown above the branches and bootstrap values below. Values of 100% are represented by an asterisk. Missing values indicate weakly supported nodes (pp and bootstrap < 50). Outgroup species are not shown. For locality data see Table 1 and Appendix 1.

**Figure 2.**
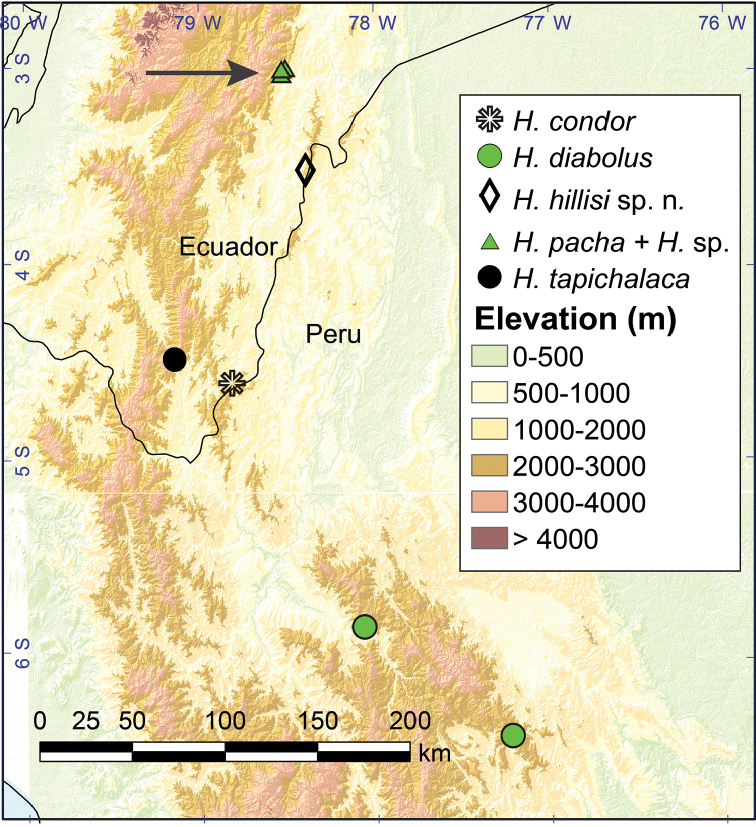
Records of the Southern Clade of the *Hyloscirtuslarinopygion* group. Locality data were obtained from specimens deposited at Museo de Zoología, Pontificia Universidad Católica del Ecuador (QCAZ), Duellman and Hillis (1990), Almendáriz et al. (2014a), and Rivera-Correa et al. (2016). The arrow indicates the locality where the Northern and Southern clades are sympatric. See text for details.

**Figure 3.**
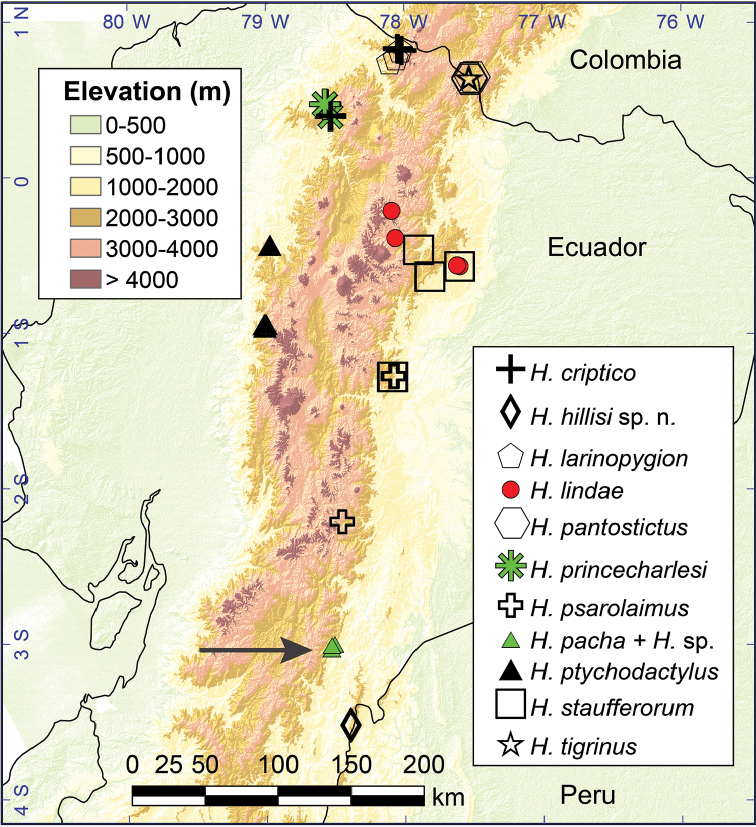
Records of the Northern Clade of the *Hyloscirtuslarinopygion* group. Locality data were obtained from specimens deposited at Museo de Zoología, Pontificia Universidad Católica del Ecuador (QCAZ) and Duellman and Hillis (1990). The arrow indicates the locality where the Northern and Southern clades are sympatric. See text for details.

According to PartitionFinder, the best partition strategy consisted of two partitions under model GTR + I + G. Maximum likelihood and Bayesian inference analyses resulted in similar topologies. Four species groups within *Hyloscirtus* (*H.jahni, H.bogotensis, H.armatus*, and *H.larinopygion* group) were recovered with strong support (posterior probability, pp = 1.0 and bootstrap = 100) in both analysis (Figure 1). However, phylogenetic relationships among these groups were weakly supported (pp < 0.71 and bootstrap < 50), as previously reported (Almendáriz et al. 2014; Coloma et al. 2012; Guayasamin et al. 2015; Rojas-Runjaic et al. 2018). The only exception was the strong support found for the clade *H.armatus* group + *H.larinopygion* group found in the Bayessian analysis (pp = 0.99). The phylogeny shows *Hyloscirtushillisi* sp. n. sister to *Hyloscirtus* sp. + *H.tapichaca*. *Hyloscirtus* sp. (KU 202728) is an undescribed species previously referred as “*H.lindae*” (Almendáriz et al. 2014; Duellman and Hillis 1990).*Hyloscirtuscondor* is sister to a clade conformed by these three species. All together form a strongly supported clade distributed in the eastern slopes of the Andes of central and southern Ecuador and northern Peru (Southern Clade; Figs 1, 2). The Southern Clade is sister to a clade distributed to the north and confirmed by the remaining species of the *Hyloscirtuslarinopygion* group (Northern Clade; Figs 1, 3). The Northern and Southern clades have a narrow zone of sympatry in central Ecuador (Figure 2).

Genetic distances between the new species and its closest relatives are characteristic of interspecific distances for the *H.larinopygion* group. For gene 12S, distances with *H.tapichalaca* are 0.031 to 0.038 and with *H.* sp. (KU 202728) are 0.031 to 0.033. These distances are higher than those observed for the same gene between *H.pacha* and *H.staufferorum* (0.014–0.018), *H.princecharlesi* and *H.ptychodactylus* (0.004–0.020) and *H.criptico* and *H.psarolaimus* (0.022–0.026; Almendáriz et al. 2014). Genetic distances for gene 16S range from 0.029 and 0.040 (Table 2). The genetic divergence between *H.hillisi* sp. n. and its closest relatives and its unique morphology indicates that it is a new species that we describe below.

**Table 2.** Pairwise genetic distances (uncorrected-*p*) between *Hyloscirtushillisi* sp. n. and its closest relatives, based on sequences of 16S mtDNA. Mean and ± standard deviation are given with range in parentheses. Diagonal values are intraspecific distances.

	*H.hillisi* sp. n. (*n* = 5)	*H.tapichalaca* (*n* = 2)	*H.condor* (*n* = 2)
*H.hillisi* sp. n.	0.001 ± 0.0007 (0–0.002)	–	–
* H. tapichalaca *	0.029 ± 0.0005 (0.029–0.030)	0.009	–
* H. condor *	0.04 ± 0.0005 (0.039–0.040)	0.041 ± 0.002 (0.039–0.043)	0

#### Hyloscirtus hillisi sp. n.

Taxon classificationAnimaliaAnuraHylidae

http://zoobank.org/95C54DD9-297E-471D-8E5F-2B96BE740147

##### Holotype.

QCAZ 68649 (Figs 5–7), field no. SC 59176, adult female from Ecuador, Provincia Morona Santiago, Caverns-cascade trail, Reserva Biológica El Quimi, on the slopes of flat-topped mountain on the eastern side of the Río Quimi valley (3.5190S, 78.3788W), 2128 m above sea level, collected by Diego Almeida, Darwin Núñez, Kunam Nucirquia, Alex Achig, and Ricardo Gavilanes on 8 July 2017.

##### Paratopotypes.

QCAZ 68646, 72549 subadult females, 68651–54, 72552, tadpoles, 69001, metamorphs, 72550, 72553, adult males, 2112–2134 m of elevation. Collected on 7–14 July 2017 and 12–19 April 2018 by Diego Almeida, Darwin Núñez, Kunam Nucirquia, Alex Achig, Ricardo Gavilanes, and María del Mar Moretta.

##### Paratypes.

All specimens from Reserva Biológica el Quimi, eastern side of the Río Quimi valley, Provincia Morona Santiago, Ecuador. Base camp surroundings, near Río Cristalino (3.5183S, 78.3914W), 1992 m, QCAZ 68647, juvenile, 68648, 68650, metamorphs, 68655–56, 71182, tadpoles collected on 4, 8–9 July 2017; second plateau, near limestone cave (3.5189S, 78.3815W), 2121 m, QCAZ 72551, adult male, collected on 19 April 2018. Collected by Diego Almeida, Darwin Núñez, Kunam Nucirquia, Alex Achig, and Ricardo Gavilanes.

##### Diagnosis.

The diagnosis and comparisons are based on one adult female, three adult males, and two subadult females. The new species is diagnosed by the following characters: mean SVL 70.3 mm in adult males (range 66.7–72.3; n = 3), 65.8 mm in one adult female; vomerine odontophores conic-shaped with a gap medially, each process with three to five prominent teeth; supracloacal flap ill-defined; supratympanic fold present; finger webbing formula: I basal II2-—3-III2½—2IV, toe webbing formula: I2-—2II1^+^—2^+^III1½—2½IV2½—1^+^V; forelimbs hypertrophied in males; enlarged and curved prepollex protruding as a spine in both sexes; fleshy calcar absent; dorsum, flanks, and dorsal areas of limbs dark grayish brown with tiny orange marks varying from abundant to sparse; venter dark grayish brown; iris bronze or yellowish with dark brown reticulation.

##### Comparisons.

**Figure 4.**
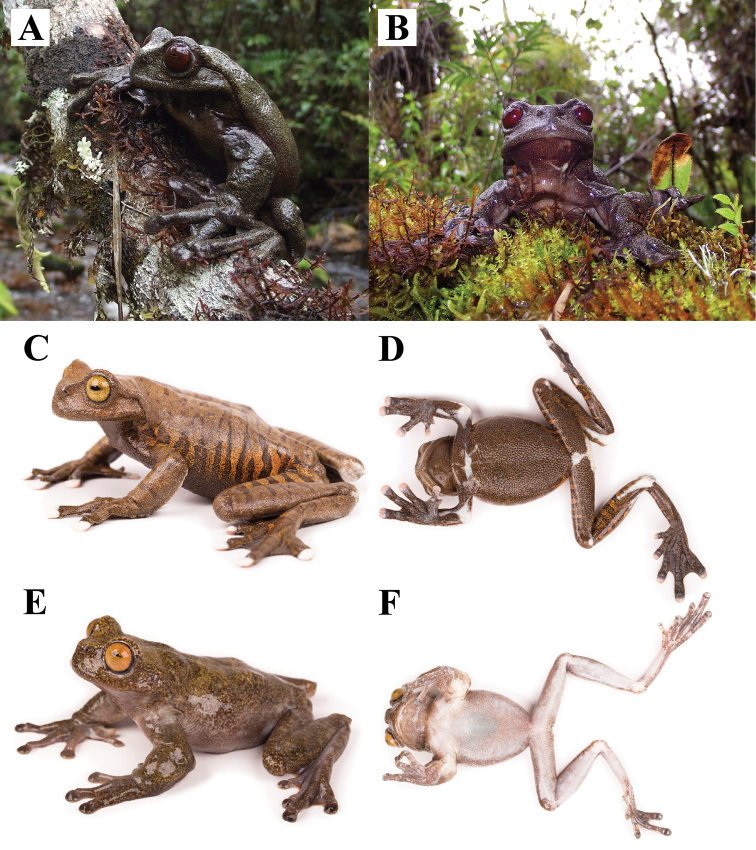
Live individuals of *Hyloscirtus*. **A, B***Hyloscirtusdiabolus* (CORBIDI 12885, adult male, holotype, SVL = 82.3 mm); **C, D***H.tapichalaca* (QCAZ 63872, adult female, SVL = 76.19 mm); **E, F***H.condor* (QCAZ 65237, adult male, SVL = 67.18 mm). Photographs: Karla García-Burneo, Diego Quirola, and Santiago Ron.

*Hyloscirtushillisi* is most similar to *H.condor*, *H.diabolus*, and *H.tapichalaca* (Figure 4). They share the presence of an enlarged claw-like prepollex. *Hyloscirtuscondor* differs in ventral coloration (light gray to light salmon in *H.condor* vs. dark brown in *H.hillisi*) and dorsal coloration (brown dorsum with diffuse yellow speckling in *H.condor* vs. dark brown dorsum with contrasting orange round marks in *H.hillisi*). *Hyloscirtusdiabolus* differs from *H.hillisi* by having a red iris (bronze or yellowish with brown reticulations in *H.hillisi*) and a fleshy calcar (calcar absent in *H.hillisi*; Rivera-Correa et al. 2016). *Hyloscirtustapichalaca* differs from *H.hillisi* by having a brown dorsum without orange marks (orange marks present in *H.hillisi*) and white disks on fingers and toes (disks are dark brown in *H.hillisi*). The remaining species of the *H.larinopygion* group lack the enlarged claw-like prepollex (Ardila-Robayo et al. 1993; Mueses-Cisneros and Anganoy-Criollo 2008; Mueses-Cisneros and Perdomo-Castillo 2011; Ruiz-Carranza and Lynch 1982; Rivera-Correa et al. 2016).

##### Description of the holotype.

**Figure 5.**
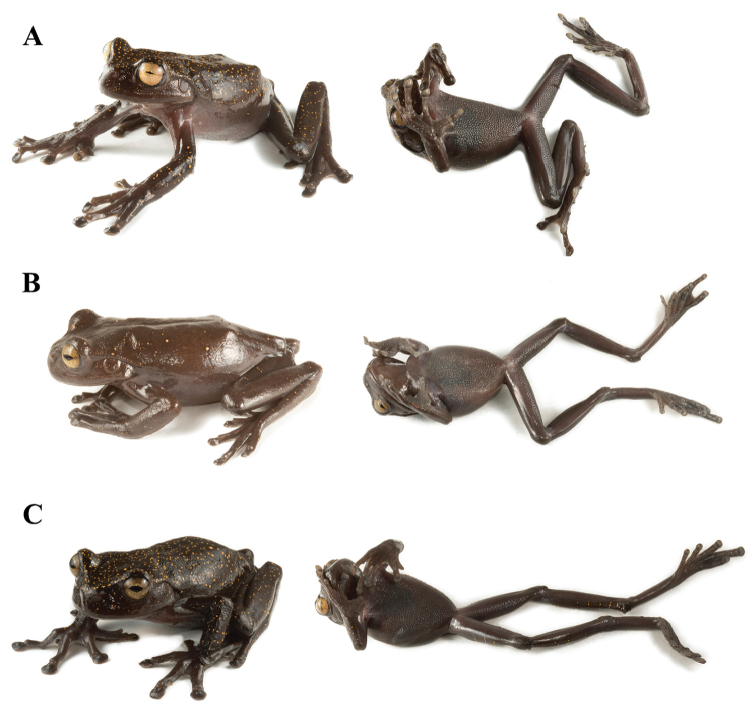
Variation in life of *Hyloscirtushillisi* sp. n. from Reserva Biológica El Quimi. **A**QCAZ 68649 (adult female, holotype, SVL = 65.78 mm) **B**QCAZ 68646 (subadult female, SVL = 48.55 mm) **C** not collected.

**Figure 6.**
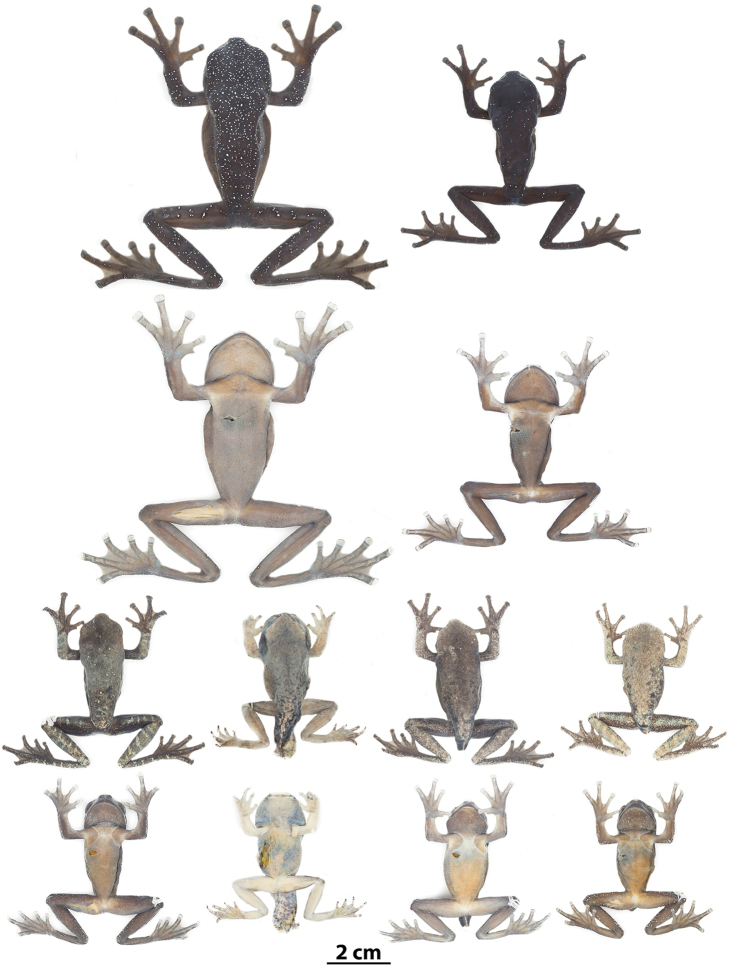
Variation of preserved specimens of *Hyloscirtushillisi* sp. n. From left to right, first and second rows: QCAZ 68649 (holotype, adult female), QCAZ 68646 (subadult female); third and fourth rows: QCAZ 68647 (juvenile), QCAZ 69001, 68650, 68648 (metamorphs).

**Figure 7.**
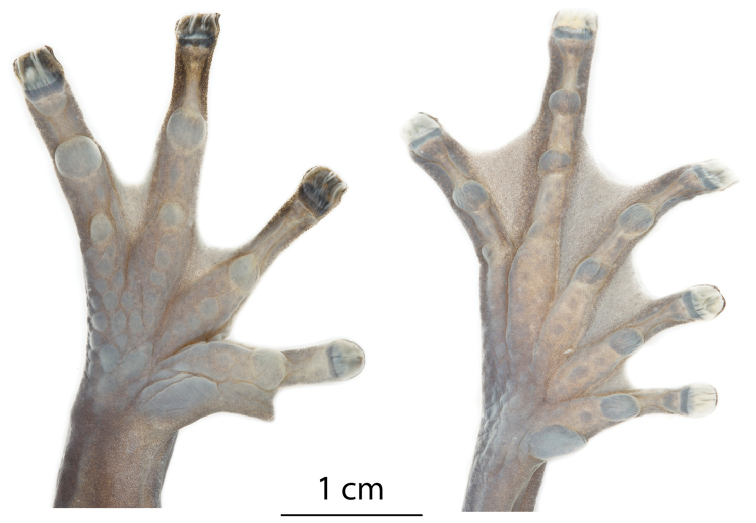
Ventral views of the left hand and foot of *Hyloscirtushillisi* sp. n. Holotype (QCAZ 68649).

An adult female (Figs 5–7), 65.78 mm SVL. Head round in dorsal view, wider than long; snout nearly truncate in lateral and dorsal views; distance from nostril to eye shorter than diameter of eye; canthus rostralis rounded; loreal region slightly concave; internarial region nearly flat; top of head slightly concave; nostrils slightly protruding anterolaterally; lips rounded, not flared; interorbital area slightly convex; eye large, protuberant; diameter of eye 1.85 times diameter of tympanic annulus; supratympanic fold thick, curved, covering posterodorsal edge of tympanum, extending from eye to posterior end of mandible and to shoulder; tympanum rounded; tympanic annulus distinct, rounded, separated from eye by ca. 1.43 times its diameter.

Forearms robust compared to upper arms but not hypertrophied; axillary membrane absent; ulnar tubercles absent; relative length of fingers I < II < IV < III; fingers bearing large, oval discs, wider than finger; subarticular tubercles prominent, round to ovoid, single; supernumerary tubercles present, small and rounded; thenar tubercle, elliptical; palmar tubercle round; prepollical tubercle large, elliptical; prepollex enlarged, claw shaped; webbing formula of fingers I basal II2-—3-III2½—2IV (Fig. 7).

Toes bearing discs broadly expanded, rounded and slightly smaller than those of fingers; relative length of toes I < II < III < V < IV; inner metatarsal tubercle large, oval; outer metatarsal tubercle absent; subarticular tubercles single, round, large, and protuberant; supernumerary tubercles present; toes webbing formula I2-—2II1^+^—2^+^III1½—2½IV2½—1^+^V (Fig. 7).

Skin on dorsum, flanks, dorsal surfaces of limbs, throat, chest, dorsal, and inner surfaces of thighs smooth; belly and ventral surfaces of thighs areolate, those of shanks smooth. Cloacal opening directed posteriorly at upper level of thighs, round tubercles below and of vent. Tongue slightly cordiform, widely attached to mouth floor; vomerine odontophores conic-shaped, separated medially, behind level of ovoid choana; each bearing 3–5 vomerine teeth. Additional measurements of the holotype are listed in Table 3.

**Table 3.** Descriptive statistics for measurements of *Hyloscirtushillisi* sp. n. Abbreviations: SVL = snout-vent length; FL = foot length; HL = head length; HW = head width; ED = eye diameter; TD = tympanum diameter; TL = tibia length; FEL = femur length. All measurements in mm.

	Adult female (holotype)	Adult males (n = 3)	Subadult females (n = 2)	Juveniles (n = 1)
SVL	65.8	70.3 ± 3.1 (66.7–72.3)	48.6–56.8	40.2
FL	29.9	30.3 ± 0.1 (30.1–30.4)	21.4–27.6	17.6
HL	14.9	14.3 ± 2.7 (11.4–16.6)	11.9–12.9	9.4
HW	22.7	24.5 ± 0.9 (23.7–25.5)	18.4–20.5	13.1
ED	6.3	6.5 ± 0.1 (6.4–6.6)	5.1–5.2	5.4
TD	3.4	4.3 ± 0.2 (4.1–4.3)	2.9–3.2	2.1
TL	32.3	33.9 ± 0.6 (33.4–34.6)	25.6–28.1	21.2
FEL	35.2	35.9 ± 1.7 (34.3–37.7)	25.7–32.36	20.9

##### Color of holotype in preservative.

(Figure 6). Dorsal surfaces of head, body, and limbs, including fingers, dark grayish-brown densely stippled with minute, cream flecks. Ventral surfaces of limbs and belly grayish-brown, ventral surfaces of discs, webbing, chest, and throat paler.

##### Color of holotype in life.

(Figure 5A). Based on digital photographs. Dorsal surfaces same as above except that flecks are bright orange. Ventral surfaces are dark grayish-brown. Ventral pads of digital discs on fingers and toes are gray. Iris is yellowish-cream.

##### Variation.

Dorsal and ventral variation of preserved individuals is depicted in Figure 6. Morphometric variation is shown in Table 3. In preservative, dorsum varies from dark grayish-brown (e.g., QCAZ 68646) in adults to pale grayish-brown (e.g., QCAZ 68647, 68650) or pale gray (e.g., QCAZ 68648) in juveniles and metamorphs. Scattered minutes cream flecks can be present on dorsal surfaces (e.g., QCAZ 68646, 68647). Specimen QCAZ 68647 (juvenile) has cream transverse bars on the dorsal surfaces of the limbs (two to four on the forearm and five to seven on the thigh, shank, and foot). Ventral surfaces vary from pale grayish-brown (e.g., QCAZ 68646) to pale brown or cream (e.g., QCAZ 68648, 68650). Coloration of webbing and discs vary from dark grayish-brown to pale grayish-brown or gray.

**Figure 8.**
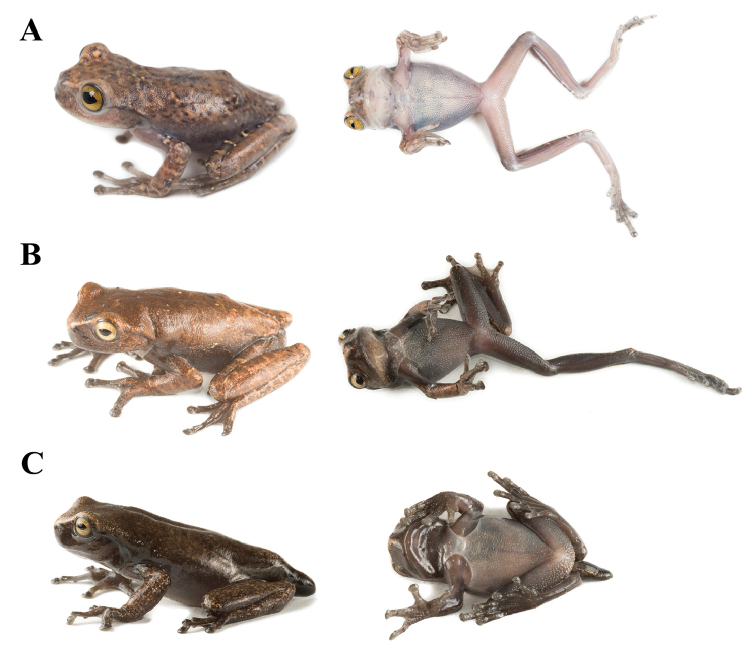
Color variation in life of juvenile and metamorphs of *Hyloscirtushillisi* sp. n. **A** SC 59268 (SVL = 39.52 mm, not preserved) **B**QCAZ 68648 (SVL = 35.6 mm) **C**QCAZ 68650 (SVL = 40.73 mm).

In life, (Figure 5), the adult specimens are very similar to the holotype except for the density of bright orange flecks (bright yellow *in situ*; Figure 11A) on the dorsal surfaces. Background dorsal coloration in juveniles and metamorphs (Figure 8) varies from mottled or uniformly brown (e.g., SC 59268, QCAZ 68650) to light brown (e.g., QCAZ 68648) with or without orange-brown transversal bars on the dorsal surfaces of the limbs. Ventral surfaces vary from dark grayish-brown to cream (e.g., SC 59268). Iris varies from bronze (e.g., SC 59268) to yellowish-cream (e.g., QCAZ 68648).

##### Tadpole description.

**Figure 9.**
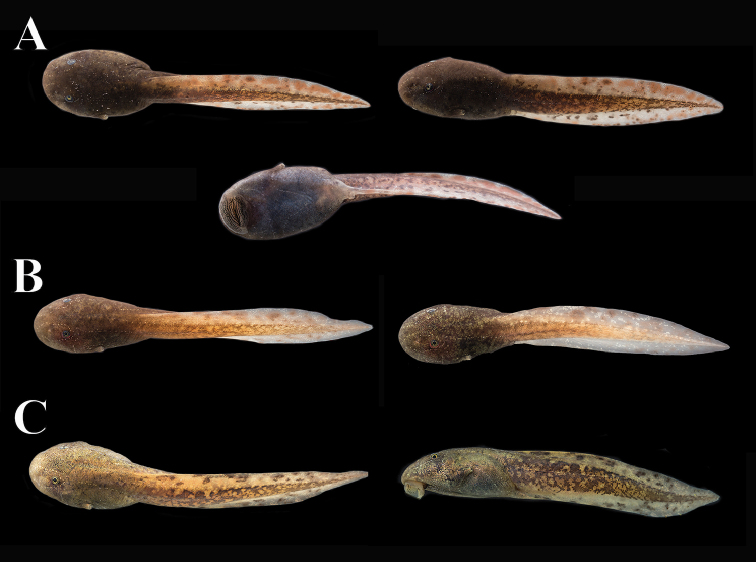
Variation in life of tadpoles of *Hyloscirtushillisi* sp. n. **A**QCAZ 68651 (photograph taken 5 days after capture on 19 July 2017) **B**QCAZ 71182 (photograph taken 16 days after capture, on 20 July 2017) **C**QCAZ 71182 (photograph taken 8 months and 4 days after capture on 08 March 2018). Note change in color between (**B**) and (**C**). Gosner Stage 25. Photographs by Gustavo Pazmiño.

The following description is based on a tadpole of series QCAZ 68651 in Stage 25 (Gosner 1960). The specimen was collected in a slow-moving pool along the margins of a stream (Figure 9; 3.5187S, 78.3919W; 1991 m) at the type locality on 7 July 2017. All measurements are in mm. Total length 86.7; body length 29.1 (33.6% of total length). Body ovoid and depressed; width at the level of spiracle 19.2, height at same position 14.7; head width at level of the eyes 17.9; anterior margin of snout uniformly rounded in dorsal view and sloping at level of nares in lateral view; lateral-line system evident with supraorbital, infraorbital, mandibular, angular, postorbital, dorsal body, and ventral body lines. The arrangement of the lateral-line system is symmetrical; the supra and infra orbital lines begin at the tip of the snout and join behind the eye, continuing as a single longitudinal line extending along the anterior half of the tail. The dorsal lines extend along the posterior half of the dorsum until reaching the anterior edge of the tail, at the base of the upper fin. The angular line starts behind the orbit and extends longitudinally, contouring the spiracle, to the posterior end of the body, down towards the venter and ending at the base of the vent tube. The postorbital line starts behind the intersection of the supra and infraorbital lines and continues obliquely towards the venter, joining the anteroventral line. The mandibular line originates at the lateral border of the oral disc and runs obliquely until joining the anteroventral line. The posteroventral line forms a V whose vertex is directed towards the midposterior venter ending at the lateral edge of the venter, at the base of the spiracle. The nostrils are ovoid, not protruding and directed anterolaterally, 6.8 from tip of snout; internarial distance 8.6. Eyes positioned and directed dorsolaterally; eye length 2.8, eye width 2.5; interorbital distance 9.9. Spiracle sinistral, located at midbody and oriented posterodorsally, inner wall free from body; tube length 2.8, tube width 2.6; spiracular opening directed posterodorsally, diameter 1.6; distance from tip of snout to spiracular opening 22.5. Vent tube medial, opening directed posteriorly; tube length 3.8, tube width 2.6. Tail length 57.5; caudal musculature robust, narrowing gradually until tail terminus. At tail-body junction, tail muscle width 9.6, tail muscle height 11.7; maximum height of tail 17.7. Oral disc located anteroventrally; transverse width 11.6; bordered by two rows of small and rounded papillae; upper jaw sheath forming an arch, unpigmented, transverse width including lateral processes 4.0 (34.4% of transverse width of oral disc); oral apparatus well preserved, showing complete teeth rows. Labial tooth row formula 8(8)/11(1). Only A-8 and P-1 have gaps. Tadpoles were gregarious and fled to the bottom of the pool when disturbed.

##### Color in preservative of tadpoles.

In dorsal view, the body is gray, lighter on the tip of snout and towards the base of the tail, grayish cream belly, mouth cream; tail musculature grayish cream with irregular gray spots, upper and lower fins transparent, light gray with irregular dark gray spots.

##### Color in life of tadpoles.

In dorsal view, body brown, including head and snout; in lateral view body dark-brown. Small bronze dots concentrate in the anterior edge of the eye, become diffuse at level of the base of the spiracle. Venter cream, becoming darker medially as result of intestines being dimly visible; oral disc light brown becoming dark brown posteriorly. Iris bronze. Vent tube cream. Muscle tail light brown with gray irregular spots; lower fin transparent cream with a combination of brown and gray irregular spots; upper fin transparent light brown with light brown spots and few scattered dark gray spots. The brown coloration and the pattern of dark gray and brown spots in several individuals is maintained; however, an individual kept in captivity (QCAZ 71182) during 8 months presents an evident change in its coloration, becoming much clearer with a combination of light brown on the back and greenish brown on the flanks; muscles of tail light brown with gray spots; lower fin cream with brown spots, upper fin greenish cream becoming transparent in the distal third with dark brown spots. The differences in coloration after 8 months in captivity may be due to the effects of diet.

##### Tadpoles variation.

**Figure 10.**
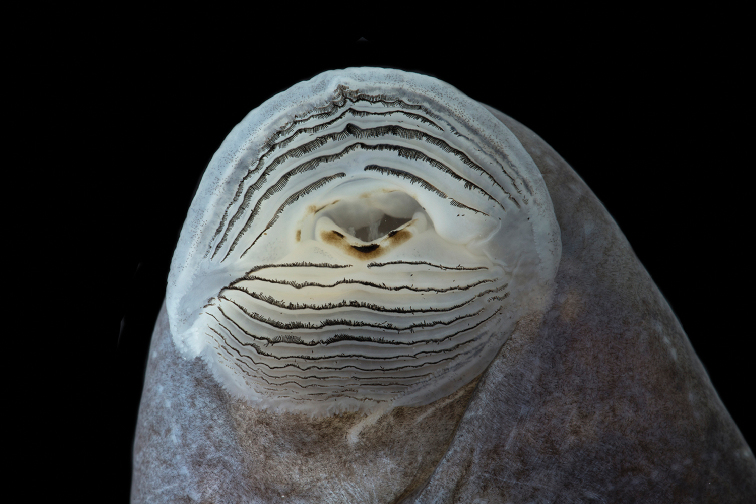
Oral disc of preserved tadpole of *Hyloscirtushillisi* sp. n. QCAZ 68651, Gosner Stage 25. Photograph by Gustavo Pazmiño.

Based on a series of five individuals in stage 25 and two in stages 37 and 40. Meristic variation of tadpoles in Stages 25–40 is shown in Table 4. Seven tadpoles in Stages 25–40 varied in total length, ranging from 57.4 to 101 mm; body length ranged from 20.4 to 34.2 mm; tail length ranged from 37.0 to 67.6 mm. Inter orbital distance from 6.27 to 10.43 mm. Labial tooth row formula varied from 8(8)/11(1) to 7(7)/12(1) (Figure 10).

**Table 4.** Measurements (in mm) of tadpoles of *Hyloscirtushillisi* sp. n. Mean ± SD is given with range in parentheses. Abbreviations: TL (total length), BL (body length), TAL (tail length), TAL/TL (ratio tail length/total length), MHT (Maximum Height of Tail, including dorsal and ventral fins), IOD (inter orbital distance), WOD (transverse width of oral disc), WUJ (transverse width of upper jaw sheath, including lateral processes), WUJ/WOD (ratio width of upper jaw sheath/width of oral disc), TUW (tube transverse width), TUL (tube length spiracle).

Character	Stage 25 (*n* = 5)	Stage 37 (*n* = 1)	Stage 40 (*n* = 1)
TL	79.2 ± 12.4 (57.4–86.7)	99.5	101
BL	26.1 ± 3.6 (20.4–29.1)	34.2	33.4
TAL	53 ± 9.02 (37–58)	65.3	67.6
TAL/TL	0.7 ± 0.04 (0.6–0.7)	0.7	0.7
MHT	15.4 ± 1.7 (13.7–17.7)	19	19.4
IOD	8.4 ± 1.5 (6.3–9.9)	10.2	10.4
WOD	9.3 ± 1.7 (7–11.6)	11.7	11.7
WUJ	3.9 ± 0.1 (3.8–4)	5.2	5.5
WUJ/WOD	2.8 ± 0.4 (2.3–3.3)	2.2	2.1
TUW	1.9 ± 0.5 (1.4–2.6)	2.7	3.6
TUL	2.4 ± 0.4 (1.7–2.8)	3.2	4.3

##### Etymology.

The specific name is a noun in the genitive case and is a patronym for David Hillis, an evolutionary biologist who has made significant contributions to the study of the evolution of amphibians and reptiles. During the 1980s, David Hillis carried out fieldwork in Ecuador that resulted in the discovery of three undescribed species of the *H.larinopygion* group. In 1990, in collaboration with WE Duellman, he published the first phylogeny for the *H.larinopygion* group using allozyme data (Duellman and Hillis 1990). Currently he is professor at the University of Texas in Austin.

##### Distribution and natural history.

**Figure 11.**
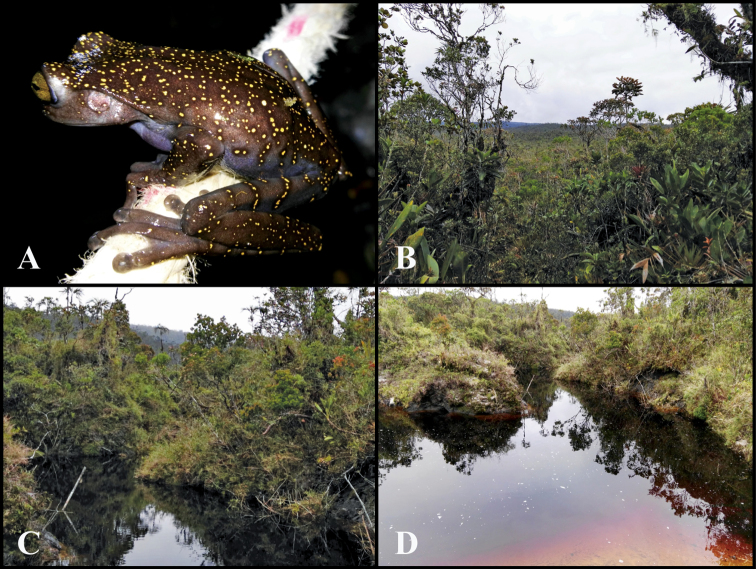
Habitat of *Hyloscirtushillisi* sp. n. **A***Hyloscirtushillisi* sp. n. *in situ***B** vegetation at the type locality, Reserva Biológica El Quimi, Ecuador **C** habitat where the adults were found **D** habitat where the tadpoles and metamorphs were found. Photographs by Diego Almeida.

*Hyloscirtushillisi* is only known from two nearby sites (airline distance = 1.7 km) on the slopes of a flattop limestone mountain in the Río Quimi basin, Provincia Zamora Chinchipe, at elevations between 1991 and 2134 m (Figure 2). Biogeographic region is Eastern Montane Forest according to Ron et al. (2018) classification. Vegetation at the type locality (Figure 11B, C) was dominated by shrubs (1.5 m tall) with sparse trees (10–15 m tall). The ground had cushioned consistency and was covered by roots and bare soil. Mosses and ground-bromeliads were abundant. This type of ground cover is locally known as *bamba*. Two adults and one juvenile were found on shrubs next to small streams on the Río Cristalino basin, at an elevation of 2134 m. The tadpoles and juveniles were found in ponds on the margin of Río Cristalino, at an elevation of 1991 m (Figure 11D). Collections took place in July 2017 and April 2018. The site where the adults were collected is ~500 m from the border between Peru and Ecuador. Therefore, the occurrence of *H.hillisi* in Peru is almost certain.

##### Conservation status.

*Hyloscirtushillisi* is only known from two nearby sites in Cordillera del Cóndor. Population size is unknown, but the scant evidence suggests low abundances. In 2017, at the site where the tadpoles and juveniles were found, five hours of nocturnal search by five experienced herpetologists yielded no adults. At the site where the adults were found, ten hours of nocturnal search, for two nights, by two experienced herpetologists, yielded two adults and one subadult. Habitat destruction and fragmentation is evident at a distance of 3.5 km from one of the collection sites (according to Ministerio de Ambiente del Ecuador 2013 map). Cordillera del Cóndor is threatened by large and small-scale mining which has already affected amphibian populations (Valencia et al. 2017). Because of its small known distribution and nearby habitat destruction and mining activities, we suggest to assign *H.hillisi* to the Critically Endangered category under criteria B1a, b(iii), according to IUCN (2001) guidelines.

## Discussion

Our phylogeny is consistent with previous phylogenies of *Hyloscirtus* (e.g., Almendáriz et al. 2014; Coloma et al. 2012; Faivovich et al. 2005; Rojas-Runjaic et al. 2018). The sister clade of the *H.larinopygion* group appears to be the *H.armatus* group (e.g., Rojas-Runjaic et al. 2018, Duellman et al. 2016, herein). A close relationship between the *H.armatus* group and *H.larinopygion* group is also supported by the shared presence of an enlarged prepollex protruding as a spine in the *H.armatus* group and in the Southern Clade of the *H.larinopygion* group. Under Duellman et al. (2016) topology, the absence of the spine in the Northern Clade would result from a secondary loss.

*Hyloscirtushillisi* is the second species of the *Hyloscirtuslarinopygion* group to be discovered in Cordillera del Cóndor, a sub-Andean mountain chain with unique geology. While the main Andes are composed of igneous and metamorphic rocks, Cordillera del Cóndor is composed predominantly by sedimentary rocks, specially limestone and sandstone (Neill 2005). Although much younger, Cordillera del Cóndor is geologically similar to the Tepuis in the Guianan region. Remarkably, surveys of the plant communities of Cordillera del Cóndor have recorded at least 10 genera that otherwise are endemic or nearly endemic to the Guianan Tepuis (Ulloa and Neill 2006).

The biogeographic affinity between the biotas of Cordillera del Cóndor and the Guianan Tepuis can be tested with phylogenies. Close relationships between biotas from El Cóndor and the Guianan Tepuis are expected under that biogeographic scenario. However, a review of recently published phylogenies is inconsistent with a Cóndor-Guianan link. Our phylogeny, for example, shows that both species of *Hyloscirtus* from el Cóndor are closely related to Andean species from southern Ecuador and northern Peru. Similar results are evident in *Pristimantismuranunka* (closely related to *Pristimantis* from the Andes of southern Ecuador; Brito et al. 2017), *Pristimantisyantzaza* (closely related to *Pristimantis* from the Andes and adjacent Amazonian lowlands of Peru and Ecuador; Valencia et al. 2017), *Excidobatescondor* (closely related to *Excidobates* from Cordillera del Cóndor and adjacent Amazonian lowlands; Almendáriz et al. 2012), *Centrolenecondor* (sister to a large clade of *Centrolene* with species from the Andes of Venezuela, Colombia, Ecuador and Peru; Castroviejo‐Fisher et al. 2014), and *Chiasmocleisparkeri* (closely related to *Chiasmocleis* from the Amazonian lowlands; Almendáriz et al. 2017). The combined evidence indicates that the biogeographic link between Cordillera del Cóndor and the Tepui region is not discernable in amphibians.

We suspect that the difference in biogeographic pattern observed between plants and amphibians may result from differences in the ecological factors that influence their geographic distribution. In plants, a key factor is soil type (e.g., Clark et al. 1999). The similarity in soil type between Cordillera del Cóndor and the Tepui region (Neill 2005) may explain the biogeographic connection observed in plants. In amphibians, in contrast, edaphic conditions appear to be of minor importance explaining the lack of biogeographic affinity between both regions.

As result of its historic inaccessibility, the organismal diversity of Cordillera del Cóndor is poorly known. During the last two decades, after armed conflicts between Ecuador and Peru ended, roads began to be built and biodiversity surveys became more frequent. These surveys have revealed a large number of unknown species of amphibians, several of which have been recently described (e.g., Almendáriz et al. 2014; Almendáriz et al. 2017; Almendáriz et al. 2012; Almendáriz et al. 2014; Brito et al. 2017; Brito et al. 2014; Terán-Valdez and Guayasamín 2010; Valencia et al. 2017). Additional expeditions to Cordillera del Cóndor are likely to result in more discoveries since it remains largely unexplored.

## Supplementary Material

XML Treatment for Hyloscirtus hillisi

## Appendix 1

**Table:** Examined specimens. All specimens were collected in Ecuador and are deposited at the Museum of Zoology, Pontificia Universidad Católica del Ecuador (QCAZ).

Species	Museum Number	Province	Locality
* Hyloscirtus condor *	QCAZ 65235	Zamora Chinchipe	Reserva Biológica Cerro Plateado, 2200 m; 4.6045S, 78.8227W
* H. condor *	QCAZ 65236	Zamora Chinchipe	Reserva Biológica Cerro Plateado, 2243 m; 4.6044S, 78.8226W
* H. condor *	QCAZ 65237	Zamora Chinchipe	Reserva Biológica Cerro Plateado, 2219 m; 4.6044S, 78.8238W
* H. condor *	QCAZ 65240	Zamora Chinchipe	Reserva Biológica Cerro Plateado, 2320 m; 4.6050S, 78.8166W
* H. condor *	QCAZ 65241	Zamora Chinchipe	Reserva Biológica Cerro Plateado, 2320 m; 4.6050S, 78.8166W
* H. criptico *	QCAZ 4161	Carchi	22 km E Maldonado, Maldonado-Tulcán Road, 2560 m; 0.8301N, 78.0456W
* H. criptico *	QCAZ 4168	Carchi	22 km E Maldonado, Maldonado-Tulcán Road, 2560 m; 0.8301N, 78.0456W
* H. criptico *	QCAZ 4169	Carchi	22 km E Maldonado, Maldonado-Tulcán Road, 2560 m; 0.8301N, 78.0456W
* H. criptico *	QCAZ 4170	Carchi	22 km E Maldonado, Maldonado-Tulcán Road, 2560 m; 0.8301N, 78.0456W
* H. criptico *	QCAZ 10487	Imbabura	Cuellaje, 1813 m; 0.4N, 78.525W
* H. criptico *	QCAZ 11989	Carchi	22 km E Maldonado, Maldonado-Tulcán Road, 2560 m; 0.8260N, 78.0420W
* H. criptico *	QCAZ 41467	Imbabura	Seis de Julio de Cuellaje, 2800 m; 0.3968N, 78.5273W
* H. criptico *	QCAZ 42149	Imbabura	Cuellaje, San Antonio, Reserva Ecológica Cotacachi Cayapas, 2720 m; 0.4775N, 78.5626W
* H. criptico *	QCAZ 42150	Imbabura	Cuellaje, San Antonio, Reserva Ecológica Cotacachi Cayapas, 2720 m; 0.4775N, 78.5626W
* H. criptico *	QCAZ 42152	Imbabura	Cuellaje, San Antonio, Reserva Ecológica Cotacachi Cayapas, 2720 m; 0.4775N, 78.5626W
* H. criptico *	QCAZ 42153	Imbabura	Cuellaje, San Antonio, Reserva Ecológica Cotacachi Cayapas, 2720 m; 0.4775N, 78.5626W
* H. criptico *	QCAZ 42156	Imbabura	Cuellaje, San Antonio, Reserva Ecológica Cotacachi Cayapas, 2720 m; 0.4775N, 78.5626W
* H. criptico *	QCAZ 42157	Imbabura	Cuellaje, San Antonio, Reserva Ecológica Cotacachi Cayapas, 2720 m; 0.4775N, 78.5626W
* H. criptico *	QCAZ 42168	Imbabura	Cuellaje, San Antonio, Reserva Ecológica Cotacachi Cayapas, 2720 m; 0.4775N, 78.5626W
* H. criptico *	QCAZ 43421	Imbabura	Cuellaje, San Antonio, Reserva Ecológica Cotacachi Cayapas, 2560 m; 0.4747N, 78.5550W
* H. criptico *	QCAZ 43422	Imbabura	Cuellaje, San Antonio, Reserva Ecológica Cotacachi Cayapas, 2560 m; 0.4747N, 78.5550W
* H. criptico *	QCAZ 43500	Imbabura	Cuellaje, San Antonio, Reserva Ecológica Cotacachi Cayapas, 2794 m; 0.4732N, 78.5702W
* H. criptico *	QCAZ 43503	Imbabura	Cuellaje, San Antonio, Reserva Ecológica Cotacachi Cayapas, 2830 m; 0.4758N, 78.5679W
* H. criptico *	QCAZ 43516	Imbabura	Cuellaje, San Antonio, 2760 m; 0.4724N, 78.5660W
* H. criptico *	QCAZ 43517	Imbabura	Cuellaje, San Antonio, 2760 m; 0.4724N, 78.5660W
* H. criptico *	QCAZ 43518	Imbabura	Cuellaje, San Antonio, 2765 m; 0.4724N, 78.5660W
* H. criptico *	QCAZ 43528	Imbabura	Cuellaje, San Antonio, 2885 m; 0.4724N, 78.5660W
* H. criptico *	QCAZ 44894	Imbabura	Cuellaje, San Antonio, Reserva Ecológica Cotacachi Cayapas, 2720 m; 0.4775N, 78.5626W
* H. criptico *	QCAZ 44895	Imbabura	Cuellaje, San Antonio, Reserva Ecológica Cotacachi Cayapas, 2720 m; 0.4775N, 78.5626W
* H. criptico *	QCAZ 45466	Carchi	Tulcán-Maldonado Road, Quebrada Centella, 2806 m; 0.8179N, 78.016W
* H. criptico *	QCAZ 50320	Imbabura	Seis de Julio de Cuellaje, 1858 m; 0.3968N, 78.5273W
* H. criptico *	QCAZ 57951	Imbabura	Cuellaje, San Antonio, Reserva Ecológica Cotacachi Cayapas, 2720 m; 0.4775N, 78.5626W
* H. criptico *	QCAZ 57952	Imbabura	Cuellaje, San Antonio, Reserva Ecológica Cotacachi Cayapas, 2720 m; 0.4775N, 78.5626W
* H. larinopygion *	QCAZ 29211	Carchi	24 km Maldonado, Tulcán Road, 2664 m; 0.8231N, 78.0253W
* H. larinopygion *	QCAZ 29212	Carchi	24 km Maldonado, Tulcán Road, 2664 m; 0.8231N, 78.0253W
* H. larinopygion *	QCAZ 38418	Carchi	Cerro Centella, Tulcán-Maldonado Road, 2788 m; 0.8143N, 78.0149W
* H. larinopygion *	QCAZ 41826	Carchi	Cañón de Morán, 2452 m; 0.7467N, 78.1038W
* H. larinopygion *	QCAZ 45462	Carchi	Tulcán-Tufiño-Maldonado Road, Quebrada Centella, 2806 m; 0.8179N, 78.0160W
* H. larinopygion *	QCAZ 55574	Carchi	Morán, 2800 m; 0.7729N, 78.0559W
* H. larinopygion *	QCAZ 55575	Carchi	Morán, 2800 m; 0.7729N, 78.0559W
* H. lindae *	QCAZ 7593	Napo	10 Km E Oyacachi, 2510 m; 0.2322S, 78.0072W
* H. lindae *	QCAZ 10483	Napo	Oyacachi, 3217 m; 0.2128S, 78.0876W
* H. lindae *	QCAZ 41232	Napo	Pacto Sumaco, Parque Nacional Sumaco, 2479 m; 0.5696S, 77.5941W
* H. lindae *	QCAZ 41294	Napo	Pacto Sumaco, Pabayacu, 2775 m; 0.5639S, 77.6154W
* H. lindae *	QCAZ 41295	Napo	Pacto Sumaco, Pabayacu, 2775 m; 0.5639S, 77.6154W
* H. lindae *	QCAZ 41296	Napo	Pacto Sumaco, Pabayacu, 2775 m; 0.5639S, 77.6154W
* H. lindae *	QCAZ 41297	Napo	Pacto Sumaco, Pabayacu, 2775 m; 0.5639S, 77.6154W
* H. lindae *	QCAZ 41298	Napo	Pacto Sumaco, Pabayacu, 2775 m; 0.5639S, 77.6154W
* H. lindae *	QCAZ 45342	Napo	11-12 km E Papallacta, 2700 m; 0.3884S, 78.0605W5W
* H. lindae *	QCAZ 45345	Napo	Papallacta, Papallacta-Cuyuja Road, 2600 m; 0.3884S, 78.0605W
* H. lindae *	QCAZ 45346	Napo	Papallacta, Papallacta-Cuyuja Road, 2600 m; 0.3884S, 78.0605W
* H. lindae *	QCAZ 45463	Sucumbíos	11 km S Santa Bárbara, La Bonita Road, 2341 m; 0.6159N, 77.4879W
* H. pacha *	QCAZ 10489	Morona Santiago	Gualaceo-Limón Road, 2120 m; 3.0310S, 78.5270W
* H. pacha *	QCAZ 48237	Morona Santiago	Plan de Milagro, 8 km Plan de Milagro, 2152 m; 3.0011S, 78.5052W
* H. pacha *	QCAZ 48238	Morona Santiago	Plan de Milagro, 9 km Plan de Milagro, 2300 m; 3.0079S, 78.5253W
* H. pacha *	QCAZ 48239	Morona Santiago	Plan de Milagro, 9 km Cuenca Road, 2300 m; 3.0079S, 78.5253W
* H. pacha *	QCAZ 48240	Morona Santiago	Plan de Milagro, 9 km Cuenca Road, 2300 m; 3.0079S, 78.5253W
* H. pacha *	QCAZ 48241	Morona Santiago	Plan de Milagro, 9 km Plan de Milagro, 2300 m; 3.0079S, 78.5253W
* H. pacha *	QCAZ 57944	Morona Santiago	Limón Indanza, 2300 m; 3.0079S, 78.5253W
* H. pantostictus *	QCAZ 731	Sucumbíos	3.5 km Santa Bárbara-La Bonita Road, 2690 m; 0.6490N, 77.5040W
* H. pantostictus *	QCAZ 2721	Sucumbíos	6.1 km Santa Bárbara-La Bonita Road, 2760 m; 0.6410N, 77.4989W
* H. pantostictus *	QCAZ 3753	Sucumbíos	Santa Bárbara, 2656 m; 0.6437N, 77.5257W
* H. pantostictus *	QCAZ 4505	Sucumbíos	Santa Bárbara, 2656 m; 0.6437N, 77.5257W
* H. pantostictus *	QCAZ 4506	Sucumbíos	Santa Bárbara, 2656 m; 0.6437N, 77.5257W
* H. pantostictus *	QCAZ 6596	Sucumbíos	Santa Bárbara, 2710 m; 0.6437N, 77.5257W
* H. pantostictus *	QCAZ 10661	Sucumbíos	Santa Bárbara, 2700 m; 0.64373N, 77.5257W
* H. pantostictus *	QCAZ 10671	Sucumbíos	Santa Bárbara, 2700 m; 0.6437N, 77.5257W
* H. pantostictus *	QCAZ 11660	Sucumbíos	Santa Bárbara, 2656 m; 0.6437N, 77.5257W
* H. pantostictus *	QCAZ 11661	Sucumbíos	Santa Bárbara, 2656 m; 0.6437N, 77.5257W
* H. pantostictus *	QCAZ 11662	Sucumbíos	Santa Bárbara, 2656 m; 0.6437N, 77.5257W
* H. pantostictus *	QCAZ 11663	Sucumbíos	Santa Bárbara, 2656 m; 0.6437N, 77.5257W
* H. pantostictus *	QCAZ 11664	Sucumbíos	Santa Bárbara, 2656 m; 0.6437N, 77.5257W
* H. pantostictus *	QCAZ 11665	Sucumbíos	Santa Bárbara, 2656 m; 0.6437N, 77.5257W
* H. pantostictus *	QCAZ 11666	Sucumbíos	Santa Bárbara, 2656 m; 0.6437N, 77.5257W
* H. pantostictus *	QCAZ 11667	Sucumbíos	Santa Bárbara, 2656 m; 0.6437N, 77.5257W
* H. pantostictus *	QCAZ 12171	Sucumbíos	Santa Bárbara, 2800 m; 0.6437N, 77.5257W
* H. pantostictus *	QCAZ 14084	Sucumbíos	Santa Bárbara, 2710 m; 0.6415N, 77.5218W
* H. pantostictus *	QCAZ 30529	Sucumbíos	Santa Bárbara, 2656 m; 0.6437N, 77.5257W
* H. pantostictus *	QCAZ 30530	Sucumbíos	Santa Bárbara, 2656 m; 0.6437N, 77.5257W
* H. pantostictus *	QCAZ 30531	Sucumbíos	Santa Bárbara, 2656 m; 0.6437N, 77.5257W
* H. pantostictus *	QCAZ 42350	Sucumbíos	Santa Bárbara, 2709 m; 0.6445N, 77.5228W
* H. pantostictus *	QCAZ 45435	Sucumbíos	Santa Bárbara, 2709 m; 0.6444N, 77.5522W
* H. pantostictus *	QCAZ 45438	Sucumbíos	Santa Bárbara, 2656 m; 0.6437N, 77.5257W
* H. pantostictus *	QCAZ 45440	Sucumbíos	Santa Bárbara, 2586 m; 0.6436N, 77.5323W
* H. pantostictus *	QCAZ 45449	Sucumbíos	Santa Bárbara, Quebrada Santa Bárbara, La Bonita, 2341 m; 0.6159N, 77.4879W
* H. pantostictus *	QCAZ 46587	Sucumbíos	3 km Santa Bárbara, 2600 m; 0.6328N, 77.5231W
* H. pantostictus *	QCAZ 46588	Sucumbíos	3 km Santa Bárbara, 2600 m; 0.6328N, 77.5231W
* H. princecharlesi *	QCAZ 41465	Imbabura	Seis de Julio de Cuellaje, 2800 m; 0.3968N, 78.5273W
* H. princecharlesi *	QCAZ 41466	Imbabura	Seis de Julio de Cuellaje, 2800 m; 0.3968N, 78.5273W
* H. princecharlesi *	QCAZ 42165	Imbabura	Cuellaje, San Antonio, 2720 m; 0.4775N, 78.5626W
* H. princecharlesi *	QCAZ 43654	Imbabura	Cuellaje, San Antonio, 2760 m; 0.4724N, 78.5660W
* H. princecharlesi *	QCAZ 44893	Imbabura	Cuellaje, San Antonio, Reserva Ecológica Cotacachi Cayapas, 2794 m; 0.4732N, 78.5702W
* H. psarolaimus *	QCAZ 13252	Napo	11 km SE Papallacta, 2800 m; 0.3870S, 78.0600W
* H. psarolaimus *	QCAZ 27049	Sucumbíos	Santa Bárbara, 0.8 km Julio Andrade Road, 2600 m; 0.6422N, 77.5264W
* H. psarolaimus *	QCAZ 31671	Morona Santiago	San Vicente, Parque Nacional Sangay, 15 km Lagunas de Atillo, 2815 m; 2.2102S, 78.4487W
* H. psarolaimus *	QCAZ 46095	Napo	60 km E Salcedo, 2748 m; 0.9709S, 78.2413W
* H. psarolaimus *	QCAZ 46096	Napo	60 km E Salcedo, 2748 m; 0.9709S, 78.2413W
* H. psarolaimus *	QCAZ 46097	Napo	60 km E Salcedo, 2748 m; 0.9709S, 78.2413W
* H. psarolaimus *	QCAZ 46098	Napo	60 km E Salcedo, 2748 m; 0.9709S, 78.2413W
* H. pantostictus *	QCAZ 46808	Sucumbíos	Santa Bárbara, El Corazón, 2670 m; 0.6437N, 77.5321W
* H. pantostictus *	QCAZ 46811	Sucumbíos	Santa Bárbara, 2589 m; 0.6437N, 77.5321W
* H. psarolaimus *	QCAZ 46890	Napo	Salcedo-Tena Road, km 60, 2748 m; 0.9719S, 78.2413W
* H. pantostictus *	QCAZ 46894	Sucumbíos	Santa Bárbara, 2709 m; 0.6445N, 77.5522W
* H. pantostictus *	QCAZ 46896	Sucumbíos	Santa Bárbara, 2709 m; 0.6445N, 77.5522W
* H. pantostictus *	QCAZ 46929	Sucumbíos	Santa Bárbara, 2709 m; 0.6445N, 77.5228W
* H. pantostictus *	QCAZ 50358	Sucumbíos	Santa Bárbara, 2589 m; 0.6437N, 77.5321W
* H. pantostictus *	QCAZ 50389	Sucumbíos	Santa Bárbara, 2589 m; 0.6437N, 77.5321W
* H. pantostictus *	QCAZ 50390	Sucumbíos	Santa Bárbara, 2586 m; 0.6436N, 77.5323W
* H. pantostictus *	QCAZ 50415	Sucumbíos	Santa Bárbara, 2709 m; 0.6445N, 77.5228W
* H. psarolaimus *	QCAZ 66563	Pastaza	Reserva Comunitaria Ankaku, Parque Nacional Llanganates, 2165 m; 1.2752S, 78.0657W
* H. psarolaimus *	QCAZ 66564	Pastaza	Reserva Comunitaria Ankaku, Parque Nacional Llanganates, 2315 m; 1.2764S, 78.0759W
* H. psarolaimus *	QCAZ 66565	Pastaza	Reserva Comunitaria Ankaku, Parque Nacional Llanganates, 2334 m; 1.2771S, 78.0768W
* H. psarolaimus *	QCAZ 66566	Pastaza	Reserva Comunitaria Ankaku, Parque Nacional Llanganates, 2322 m; 1.2767S, 78.0763W
* H. psarolaimus *	QCAZ 66568	Pastaza	Reserva Comunitaria Ankaku, Parque Nacional Llanganates, 2216 m; 1.2770S, 78.0698W
* H. ptychodactylus *	QCAZ 46030	Cotopaxi	Pilaló, Quebrada 2, 2500 m; 0.9424S, 78.9956W
* H. ptychodactylus *	QCAZ 46031	Cotopaxi	Pilaló, Quebrada 2, 2500 m; 0.9424S, 78.9956W
* H. staufferorum *	QCAZ 3701	Napo	Volcán Sumaco, Lago Sumaco, 2463 m; 0.5689S, 77.5948W
* H. staufferorum *	QCAZ 3704	Napo	Codillera de Guacamayos, 31 km Baeza, Archidona Road, 2210 m; 0.6505S, 77.7907W
* H. staufferorum *	QCAZ 3705	Napo	Baeza, 2040 m; 0.4634S, 77.8915W
* H. staufferorum *	QCAZ 3706	Napo	Baeza, 2040 m; 0.4634S, 77.8915W
* H. staufferorum *	QCAZ 11150	Napo	13.4 km S Río Cosanga, 2040 m; 0.6560S, 77.9129W
* H. staufferorum *	QCAZ 36278	Napo	Volcán Sumaco, Lago Sumaco, 2470 m; 0.5689S, 77.5948W
* H. staufferorum *	QCAZ 36279	Napo	Volcán Sumaco, Lago Sumaco, 2470 m; 0.5689S, 77.5948W
* H. staufferorum *	QCAZ 45962	Pastaza	Reserva Comunitaria Ankaku, Río Challuwa Yacu, Parque Nacional Llanganates, 2250 m; 1.2792S, 78.0779W
* H. staufferorum *	QCAZ 45963	Pastaza	Reserva Comunitaria Ankaku, Río Challuwa Yacu, Parque Nacional Llanganates, 2250 m; 1.2792S, 78.0779W
* H. staufferorum *	QCAZ 45965	Pastaza	Reserva Comunitaria Ankaku, Río Challuwa Yacu, Parque Nacional Llanganates, 2250 m; 1.2792S, 78.0779W
* H. staufferorum *	QCAZ 45966	Pastaza	Reserva Comunitaria Ankaku, Río Challuwa Yacu, Parque Nacional Llanganates, 2250 m; 1.2792S, 78.0779W
* H. staufferorum *	QCAZ 45967	Pastaza	Reserva Comunitaria Ankaku, Río Challuwa Yacu, Parque Nacional Llanganates, 2250 m; 1.2792S, 78.0779W
* H. staufferorum *	QCAZ 56807	Pastaza	Reserva Comunitaria Ankaku, Río Challuwa Yacu, Parque Nacional Llanganates, 2250 m; 1.2792S, 78.0779W
* H. staufferorum *	QCAZ 64480	Pastaza	Reserva Comunitaria Ankaku, Río Challuwa Yacu, Parque Nacional Llanganates, 2250 m; 1.2792S, 78.0779W
* H. staufferorum *	QCAZ 66567	Pastaza	Reserva Comunitaria Ankaku, Parque Nacional Llanganates, 2434 m; 1.2799S, 78.0826W
* H. tapichalaca *	QCAZ 15083	Zamora Chinchipe	Yangana-Valladolid Road, Reserva Tapichalaca, 2625 m; 4.4816S, 79.1491W
* H. tapichalaca *	QCAZ 15084	Zamora Chinchipe	Yangana-Valladolid Road, Reserva Tapichalaca, 2625 m; 4.4816S, 79.1491W
* H. tapichalaca *	QCAZ 15085	Zamora Chinchipe	Yangana-Valladolid Road, Reserva Tapichalaca, 2625 m; 4.4816S, 79.1491W
* H. tapichalaca *	QCAZ 16704	Zamora Chinchipe	Yangana-Valladolid Road, Reserva Tapichalaca, 2697 m; 4.4816S, 79.1491W
* H. tapichalaca *	QCAZ 16705	Zamora Chinchipe	Yangana-Valladolid Road, Reserva Tapichalaca, 2697 m; 4.4816S, 79.1491W
* H. tapichalaca *	QCAZ 16706	Zamora Chinchipe	Yangana-Valladolid Road, Reserva Tapichalaca, 2697 m; 4.4816S, 79.1491W
* H. tapichalaca *	QCAZ 17776	Zamora Chinchipe	Yangana-Valladolid Road, Reserva Tapichalaca, 2697 m; 4.4816S, 79.1491W
* H. tapichalaca *	QCAZ 17777	Zamora Chinchipe	Yangana-Valladolid Road, Reserva Tapichalaca, 2697 m; 4.4816S, 79.1491W
* H. tapichalaca *	QCAZ 46887	Zamora Chinchipe	Reserva Tapichalaca, 1637 m; 4.4730S, 79.1930W
* H. tapichalaca *	QCAZ 63872	Zamora Chinchipe	Parque Nacional Podocarpus, Tapichalaca, 2605 m; 4.4876S, 79.1479W
* H. tigrinus *	QCAZ 31550	Sucumbíos	Santa Bárbara, El Corazón, 2620 m; 0.6437N, 77.5321W
* H. tigrinus *	QCAZ 40331	Sucumbíos	Santa Bárbara, 2638 m; 0.6437N, 77.5321W
* H. tigrinus *	QCAZ 41351	Sucumbíos	0.7 km SW Santa Bárbara, Quebrada El Corazón, 2638 m; 0.6437N, 77.5321W
